# Cellulolytic *Bacillus cereus* produces a variety of short-chain fatty acids and has potential as a probiotic

**DOI:** 10.1128/spectrum.03267-23

**Published:** 2024-03-05

**Authors:** Yixiao Liao, Shihui Wu, Guixian Zhou, Shihui Mei, Zemin Yang, Shuang Li, Zhengyu Jin, Yongjun Deng, Ming Wen, Ying Yang

**Affiliations:** 1 College of Animal Science, Guizhou University, Guiyang, China; 2 Institute of Animal Diseases, Guizhou University, Guiyang, China; 3 Engineering Research Center of Animal Biological Products, Guiyang, China; Institute of Microbiology, Chinese Academy of Sciences, China

**Keywords:** potential probiotics, cellulolytic bacteria, whole-genome sequencing, short-chain fatty acids, safety assessment

## Abstract

Cellulolytic bacteria ferment dietary fiber into short-chain fatty acids, which play an important role in improving fiber utilization and maintaining intestinal health. Safe and effective cellulolytic bacteria are highly promising probiotic candidates. In this study, we isolated three strains of *Bacillus cereus,* which exhibited cellulolytic properties, from Kele pig feces. To assess the genetic basis of cellulose degradation by the isolates, whole-genome sequencing was used to detect functional genes associated with cellulose metabolism. Subsequently, we identified that the *B. cereus* CL2 strain was safe in mice by monitoring body weight changes, performing histopathologic evaluations, and determining routine blood indices. We next evaluated the biological characteristics of the CL2 strain in terms of its growth, tolerance, and antibiotic susceptibility, with a focus on its ability to produce short-chain fatty acids. Finally, the intestinal flora structure of the experimental animals was analyzed to assess the intestinal environment compatibility of the CL2 strain. In this study, we isolated a cellulolytic *B. cereus* CL2, which has multiple cellulolytic functional genes and favorable biological characteristics, from the feces of Kele pigs. Moreover, CL2 could produce a variety of short-chain fatty acids and does not significantly affect the diversity of the intestinal flora. In summary, the cellulolytic bacterium *B. cereus* CL2 is a promising strain for use as a commercial probiotic or in feed supplement.

**IMPORTANCE:**

Short-chain fatty acids are crucial constituents of the intestinal tract, playing an important and beneficial role in preserving the functional integrity of the intestinal barrier and modulating both immune responses and the structure of the intestinal flora. In the intestine, short-chain fatty acids are mainly produced by bacterial fermentation of cellulose. Therefore, we believe that safe and efficient cellulolytic bacteria have the potential to be novel probiotics. In this study, we systematically evaluated the safety and biological characteristics of the cellulolytic bacterium *B. cereus* CL2 and provide evidence for its use as a probiotic.

## INTRODUCTION

The World Health Organization and the Food and Agriculture Organization of the United Nations define probiotics as “live microorganisms, which when administered in adequate amounts, confer a health benefit on the host” (1). Probiotics are widely used in the commercial, medical, livestock industries and have a long history of safe use (2, 3). However, the probiotics being researched and in commercial development are from a limited list of genera, mainly *Lactobacillus* and *Bifidobacterium* (4). The developed multiomics and sequencing technologies have allowed more knowledge to be gained on the composition and function of the intestinal microbiome. Subsequently, the range of organisms with potential health benefits has been extended, and the research on probiotics has entered a new era (5–7).

Cellulose, a macromolecular polysaccharide composed of glucose, is the most abundant renewable organic substance found on Earth. Although cellulose is present in significant quantities in everyday diets, its complex structure makes its digestion difficult in the gastrointestinal tract of animals. Within the intestinal tract, cellulose is utilized solely by cellulolytic bacteria. They ferment cellulose into short-chain fatty acids (SCFAs) via the glycolytic pathway and the pentose phosphate pathway (8). Studies have shown that SCFAs serve as a primary energy source for intestinal epithelial cells and play a pivotal role in preserving the integrity of the intestinal barrier by promoting tight junction protein expression in monolayered intestinal epithelial cells and mucus production in the intestinal wall (9, 10). Concurrently, SCFAs serve as prominent anions within the intestine, effectively reducing the pH of the intestinal environment. This acidification process facilitates the proliferation of beneficial intestinal probiotics while inhibiting the reproduction of specific pathogens. Among them, propionic acid and acetic acid have been identified as key SCFAs capable of promoting the release of host antimicrobial peptides that can then exert potent antimicrobial effects (11). SCFAs play a pivotal role in modulating the inflammatory response. They interact with G protein-coupled receptors to facilitate the maturation of intestinal intrinsic lymphocytes, mitigate neutrophil recruitment to sites of inflammation, and dampen the activation of the NLRP3 inflammasome, resulting in reduced secretion of IL-1β and IL-18. SCFAs influence the activity of histone deacetylase, thereby inhibiting the NF-κB signaling pathway, promoting the secretion of IL-10, and suppressing the secretion of IL-6 to enhance the anti-inflammatory effects (12–14). In addition, SCFAs affect brain–gut axis signaling. They induce the release of glucagon-like peptide 1 (GLP-1), tyrosylated peptide (PYY), and leptin in rodent models. Specifically, GLP-1 enhances insulin secretion and improves insulin sensitivity, while PYY modulates intestinal motility, resulting in delayed gastric emptying and increased satiety, subsequently reducing food intake. Leptin, which is transmitted via the vagus nerve to the nucleus of the solitary tract, enhances energy release and inhibits the synthesis of adipocytes, thereby mitigating obesity (15, 16). Currently, research on probiotics has placed relatively little emphasis on cellulolytic properties. With an increasing number of studies highlighting the significant impact of SCFAs on intestinal health, cellulolytic bacteria have exhibited potential for use as probiotics.

Kele pigs, a semi-captive breed in Guizhou, China, have exceptional roughage tolerance, which could be attributed to their cellulolytic intestinal flora. The rich intestinal flora resources of Kele pigs hold immense economic and scientific value.^18^ In this study, we isolated three cellulolytic strains of *Bacillus cereus* from Kele pig fecal samples. Through a comprehensive approach, we evaluated the safety and biological characteristics of these strains. Our findings provide new insights into the use of cellulolytic *B. cereus* as a probiotic.

## RESULTS

### Isolation and identification of cellulolytic bacteria

**Fig 1:**
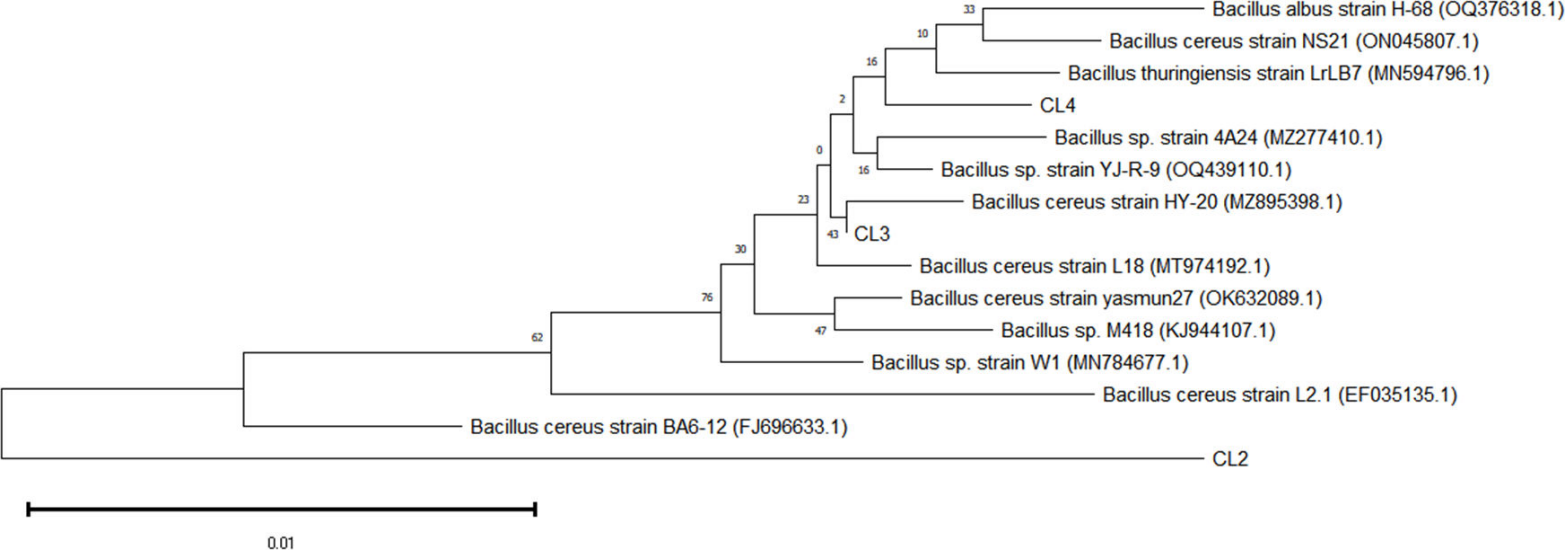
Phylogenetic tree of three *B. cereus* strains.

Three strains with high cellulase activity were isolated from the feces of Kele pigs and designated CL2 to CL4. The cellulase activities of CL2, CL3, and CL4 were 163.19 ± 3.62 U/mL, 31.49 ± 2.88 U/mL, and 87.93 ± 6.21 U/mL, respectively. Through 16S rRNA sequencing, the isolates were identified as *B. cereus*. The phylogenetic tree revealed that CL2, CL3, and CL4 formed a branch with *B. cereus* (Fig. 1) and shared 97.43%–99.24% similarity in their 16S rRNA gene sequences.

### Whole-genome sequencing and annotation

#### Gene composition and annotation

**Fig 2:**
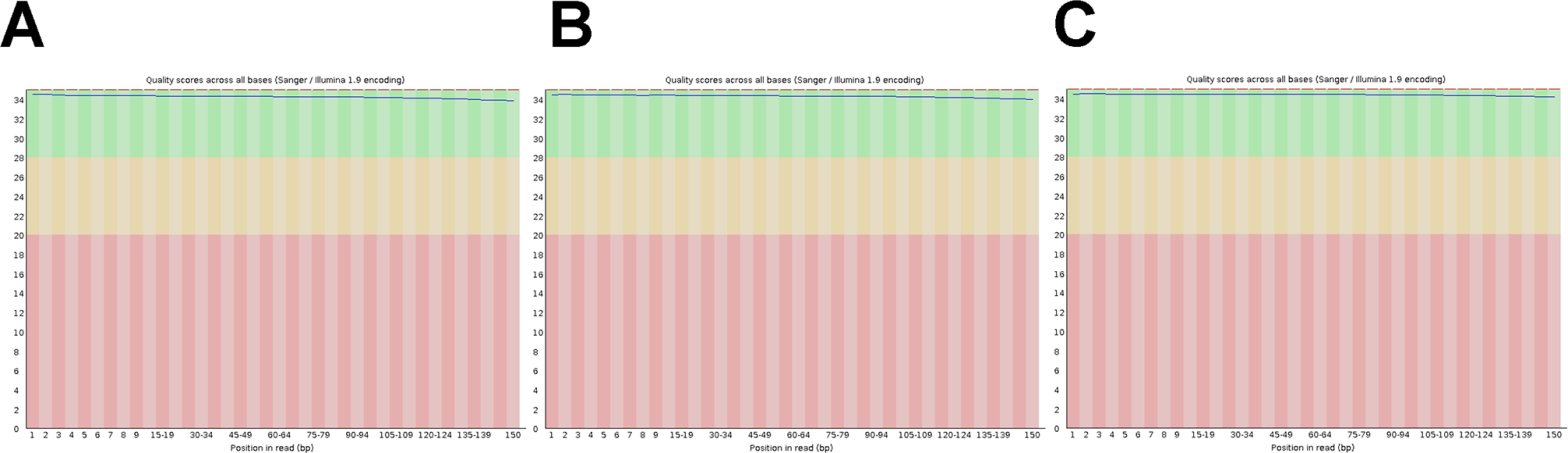
Base Mass Distribution Chart. The horizontal axis is the base position of the reads, and the vertical axis is the QC score (0–40) of all reads at that base position. Red color indicates low quality, yellow color indicates passing quality, green color indicates good quality, and the blue line is the average score. (**A**) Quality scores of the CL2 strain, (**B**) quality scores of the CL3 strain, and (**C**) quality scores of the CL4 strain.

**Fig 3:**
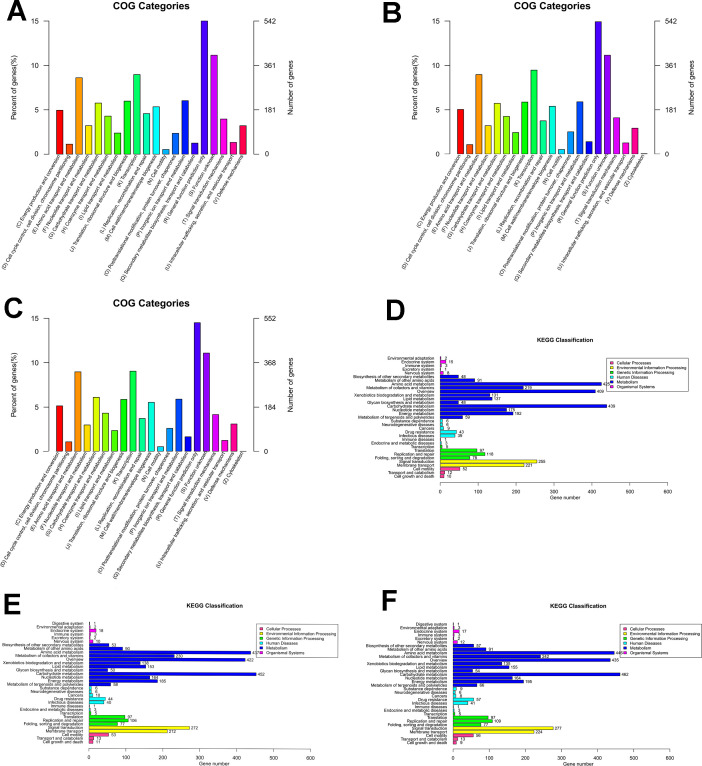
Functional gene prediction of the isolates. (**A-C**) COG annotations of isolates CL2, CL3, and CL4. (**D-F**) KEGG annotations of isolates CL2, CL3, and CL4.

Quality control revealed that whole-genome sequencing was accurate, with QC scores maintained above 30 during the process. Also, the score lines were smooth, indicating a stable sequencing process (Fig. 2). Whole-genome sequencing revealed that the *B. cereus* CL2 genes had a length of 5,446,626 bp with a GC content of 35.15%. The chromosomal sequences of CL2 were predicted to contain 5,785 coding sequences (CDSs), 13 rRNAs, and 92 tRNAs. The *B. cereus* CL3 genes had a length of 5,277,035 bp with a GC content of 35.24%. The CL3 genome was found to have 5,771 CDSs, 13 rRNAs, and 94 tRNAs. The *B. cereus* CL4 genes had a length of 5,613,809 bp with a GC content of 34.93%. The CL4 genome was predicted to have 5,776 CDSs, 13 rRNAs, and 94 tRNAs. Functional genes in the genome were annotated with COG analysis (Fig. 3A through C), and the metabolism-related genes were classified (Table 1).

**TABLE 1:** Metabolic gene classification

Predicted function	Percentage of functional genes		
	CL2	CL3	CL4
Energy production and conversion	4.95%	5.04%	5.14%
Translation, ribosomal structure, and biogenesis	5.98%	5.87%	5.87%
Amino acid transport and metabolism	8.61%	8.97%	8.97%
Carbohydrate transport and metabolism	5.76%	5.73%	6.11%
Coenzyme transport and metabolism	4.29%	4.26%	4.32%
Lipid transport and metabolism	2.38%	2.44%	2.36%
Inorganic ion transport and metabolism	6.03%	5.90%	5.90%

#### Functional gene characteristics

The results indicated that all three isolates carried multiple cellulose hydrolysis genes, including the 6-phospho-beta-glucosidase gene *celF*; the phosphotransferase system cellobiose-specific component II genes *celA*, *celB*, and *celC*; and the β-glucosidase-encoding genes *bglB* and *bglX*. Genes associated with organic acid metabolism included the acetate kinase a gene *ackA*, pyruvate kinase gene *pykF*, citrate synthase gene *gltA*, butyrate kinase gene *buk*, lactate dehydrogenase gene *ldhA*, and SCFA transport protein gene *atoE*. Table S1 provides further details. KEGG analysis revealed significant enrichment in metabolic pathways in the genomes of the isolates (Fig. 3D through F). The identified pathways included are known to be involved in the following: cellulose degradation, including the pentose phosphate pathway and glycolysis pathway; fatty acid biosynthesis, such as butanoate metabolism and propanoate metabolism; and amino acid metabolism, such as tryptophan metabolism, tyrosine metabolism, phenylalanine metabolism, and glycine metabolism. Additionally, vitamin pathways associated with thiamine metabolism, retinol metabolism, vitamin B6 metabolism, and folate biosynthesis were identified. Table S2 provides further details.

**Fig 4:**
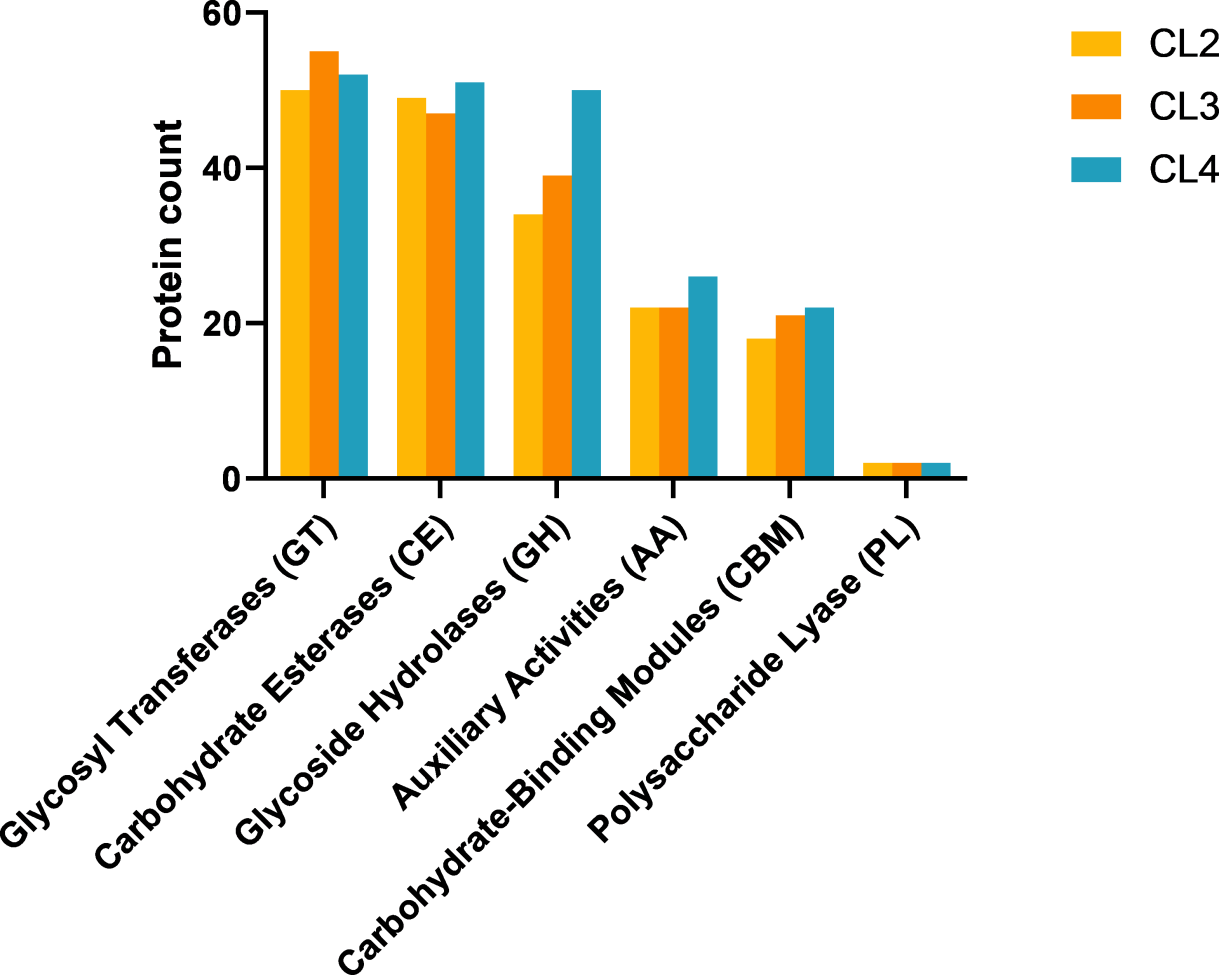
The carbohydrate-active enzyme prediction results.

The annotations of carbohydrate-active enzymes demonstrated that there were varying numbers of glycosyl transferases (GTs), carbohydrate esterases (CEs), glycoside hydrolases (GHs), auxiliary activities (AAs), carbohydrate-binding modules (CBMs), and polysaccharide lyases (PLs) in isolates CL2, CL3, and CL4 (Fig. 4). Specifically, the predicted numbers of each were as follows: GTs, 50, 55, and 52; CEs, 49, 47, and 51; GHs, 34, 39, and 50; AAs, 22, 22, and 26; CBMs, 18, 21, and 22; and PLs, 2, 2, and 2. Notably, several carbohydrase families associated with cellulose decomposition were identified, including GT2, CBM2, CBM37, CBM44, GH5, and AA10. Furthermore, isolate CL4 belonged to the CMB16 family, which is associated with cellulose decomposition. Table S3 provides further details.

Analysis of the virulence genes revealed the presence of genes associated with virulence in isolates CL2, CL3, and CL4. The three isolates all carried the cytotoxin K gene *cytK*, multiple nonhemolytic enterotoxin genes (*nheA*, *nheB*, and *nheC*), the immunosuppressant A metalloproteinase gene *inhA*, and the thiol-activated cytolysin gene *alo*. Additionally, isolates CL3 and CL4 carried the hemolysin BL genes *hblA*, *hblC*, and *hblD*. Notably, the enterotoxin FM gene was not present in the isolates.

Analysis of the resistance genes revealed that the three isolates carried β-lactam resistance genes, including *bcII*, *bcI*, and *bla1*. The rifampicin resistance gene *rpoB* and the fosfomycin resistance gene *fosB* were present in the isolates. Additionally, isolate CL3 carried the vancomycin resistance gene mutant *vanRA*, while isolate CL4 harbored the clindamycin resistance gene *lsaB* (Table 2).

**TABLE 2:** Isolates of virulence genes and resistance genes. The screening criteria were identity >80% and evaluation <1 × 10^−5*a*^

Virulence factor and drug resistance factor	Predicted genes		
	CL2	CL3	CL4
Cytotoxin K	*cytK*	*cytK*	*cytK*
Nonhemolytic enterotoxin	*nheC, nheB,* and *nheA*	*nheC, nheB,* and *nheA*	*nheC, nheB,* and *nheA*
Immune inhibitor A metalloprotease	*inhA*	*inhA*	*inhA*
Thiol-activated cytolysin	*alo*	*alo*	*alo*
Hemolysin BL	-	*hblD, hblC,* and *hblA*	*hblD, hblC,* and *hblA*
Toxin cereulide	-	-	-
Enterotoxin FM	-	-	-
β-Lactam resistance	*bcII, bcI,* and *bla1*	*bcII, bcI,* and *bla1*	*bcII, bcI,* and *bla1*
Fosfomycin resistance	*fosB*	*fosB*	*fosB*
Rifampicin resistance	*rpoB*	*rpoB*	*rpoB*
Vancomycin resistance	-	*vanRA*	-
Clindamycin resistance	-	-	*lsaB*

^*a*^ -, not detected.

### Animal safety assessment

**Fig 5:**
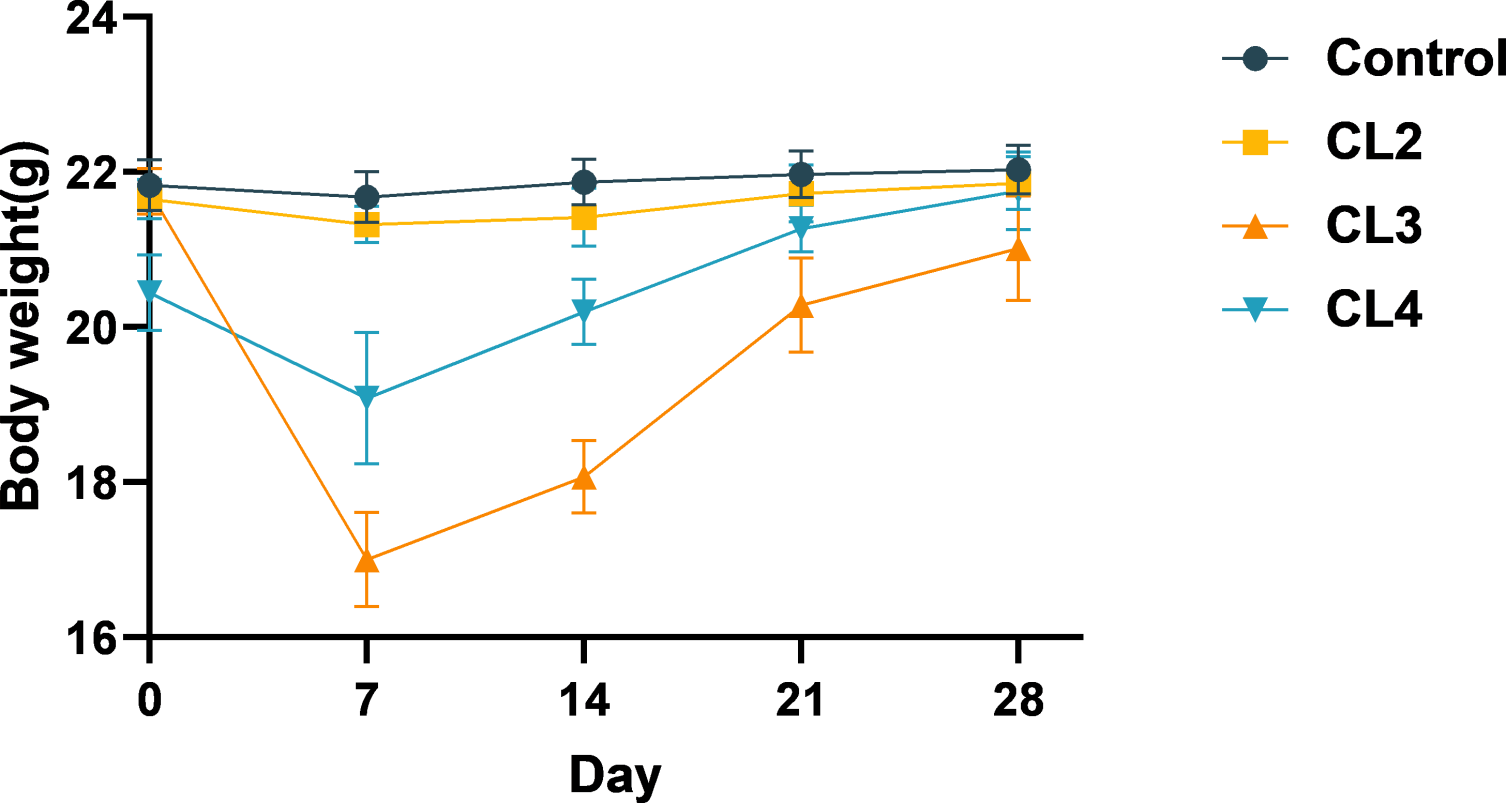
Trends in mouse body weight from days 0 to 28. The body weight of dead mice was not included in the statistics.

The safety results demonstrated that the animals in the *B. cereus* CL2 group did not exhibit significant weight loss or clinical symptoms during the 28day experimental period. Conversely, those in the *B. cereus* CL3 group and CL4 group displayed pronounced weight loss during the initial 7 days of gavage, followed by gradual recovery from days 7 to 28 (Fig. 5). Concurrently, the CL3 and CL4 groups experienced the onset of diarrhea and hematochezia, with the CL3 group showing severer symptoms. During the experiment, one mouse in the CL3 group died on day 3, two on day 5, and one on day 6. Consequently, CL2 was selected for further safety evaluations.

**Fig 6:**
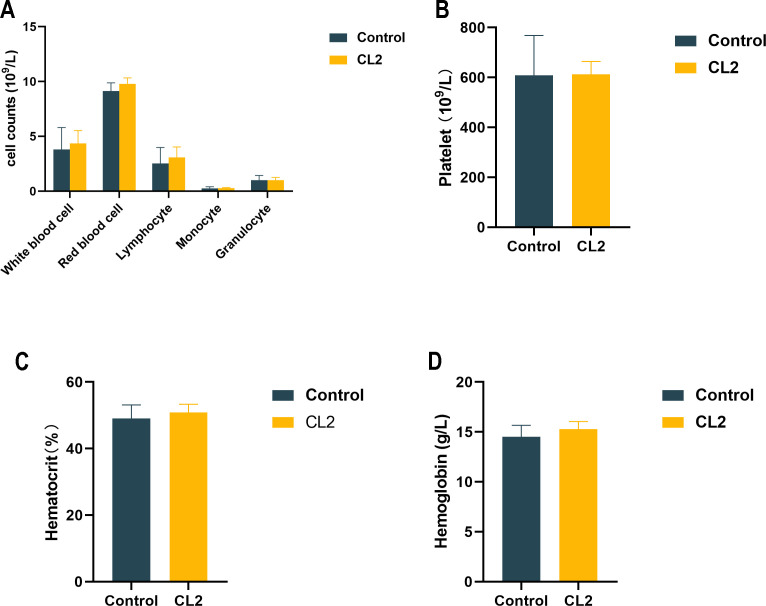
Routine blood indices of mice in the control group (*n* = 10) and the CL2 group (*n* = 10). (**A**) Blood cell counts. (**B**) Platelet counts. (**C**) Hematocrit. (**D**) Hemoglobin counts.

**Fig 7:**
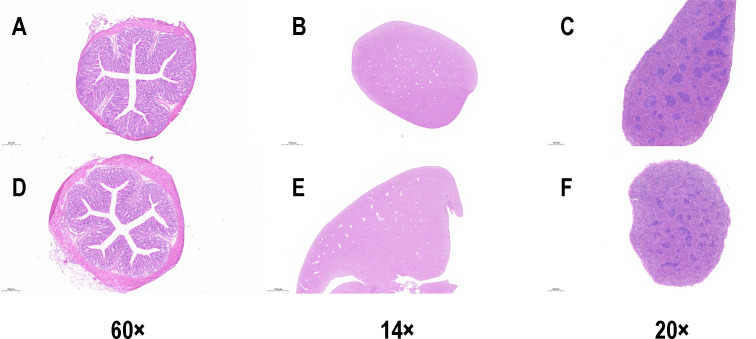
Mouse tissue sections. (**A**) Colon of a control mouse. (**B**) Liver of a control mouse. (**C**) Spleen of a control mouse. (**D**) Colon of a CL2 group mouse. (**E**) Liver of a CL2 group mouse. (**F**) Spleen of a CL2 group mouse.

Subsequent safety testing revealed no significant difference in the routine blood parameters of mice from the CL2 group (*P* > 0.05) compared to the control group on the 28th day (Fig. 6). Furthermore, no abnormalities were observed in the histological sections of the colon, spleen, and liver tissues (Fig. 7). These findings confirm that the administration of 1 × 10^8^ CFU of CL2 was nonpathogenic to mice.

### Biological characteristics

#### General characteristics

**Fig 8:**
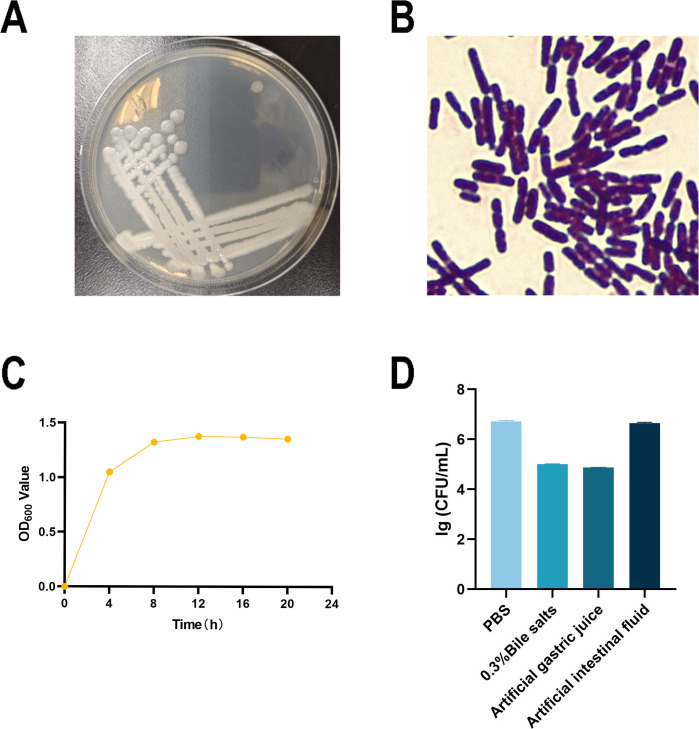
General characteristics of the isolate. (**A**) Colony morphology on the LB medium. (**B**) Gram staining characteristics. (**C**) Growth curve of the isolate. (**D**) Number of viable bacteria after isolates were placed in PBS bile salts, artificial gastric fluid, and artificial intestinal fluid for 3 h (*n* = 3).

Isolate CL2 exhibited round, protruding, irregular edges and moist, smooth, and opaque milky white colonies on the LB agar medium (Fig. 8A). It was identified as a Gram-positive *Bacillus* species (Fig. 8B) and exhibited logarithmic growth when cultured in the LB liquid medium for 4–8 h, followed by a transition to the stationary phase after 8 h (Fig. 8C). Tolerance tests revealed that CL2 was tolerant to a 0.3% bile salt environment, as well as artificial gastric and artificial intestinal fluid environments (Fig. 8D). In terms of drug susceptibility, CL2 demonstrated resistance to penicillin and ampicillin. It showed moderate susceptibility to gentamicin and novobiocin but was susceptible to streptomycin, erythromycin, azithromycin, chloramphenicol, norfloxacin, ofloxacin, clindamycin, tetracycline, and florfenicol (Table 3).

**TABLE 3:** Drug susceptibility of the isolate (*n* = 3)

Drug class	Drug	Inhibition zone diam (mm) (mean ± SD)	Result
		CL2	
β-Lactams	Penicillin	0	Resistant
	Ampicillin	0	Resistant
Aminoglycosides	Gentamycin	14.47 ± 0.56	Intermediate
	Streptomycin	17.60 ± 0.91	Susceptible
Macrolides	Erythromycin	20.13 ± 0.78	Susceptible
	Azithromycin	19.35 ± 0.72	Susceptible
	Chloramphenicol	20.63 ± 0.98	Susceptible
Quinolones	Norfloxacin	17.46 ± 0.43	Susceptible
	Ofloxacin	23.42 ± 0.77	Susceptible
Lincosamides	Clindamycin	21.87 ± 0.42	Susceptible
Tetracyclines	Tetracycline	16.23 ± 0.67	Susceptible
Others	Florfenicol	27.17 ± 1.05	Susceptible
	Novobiocin	14.99 ± 0.23	Intermediate

#### Detection of short-chain fatty acids

**Fig 9:**
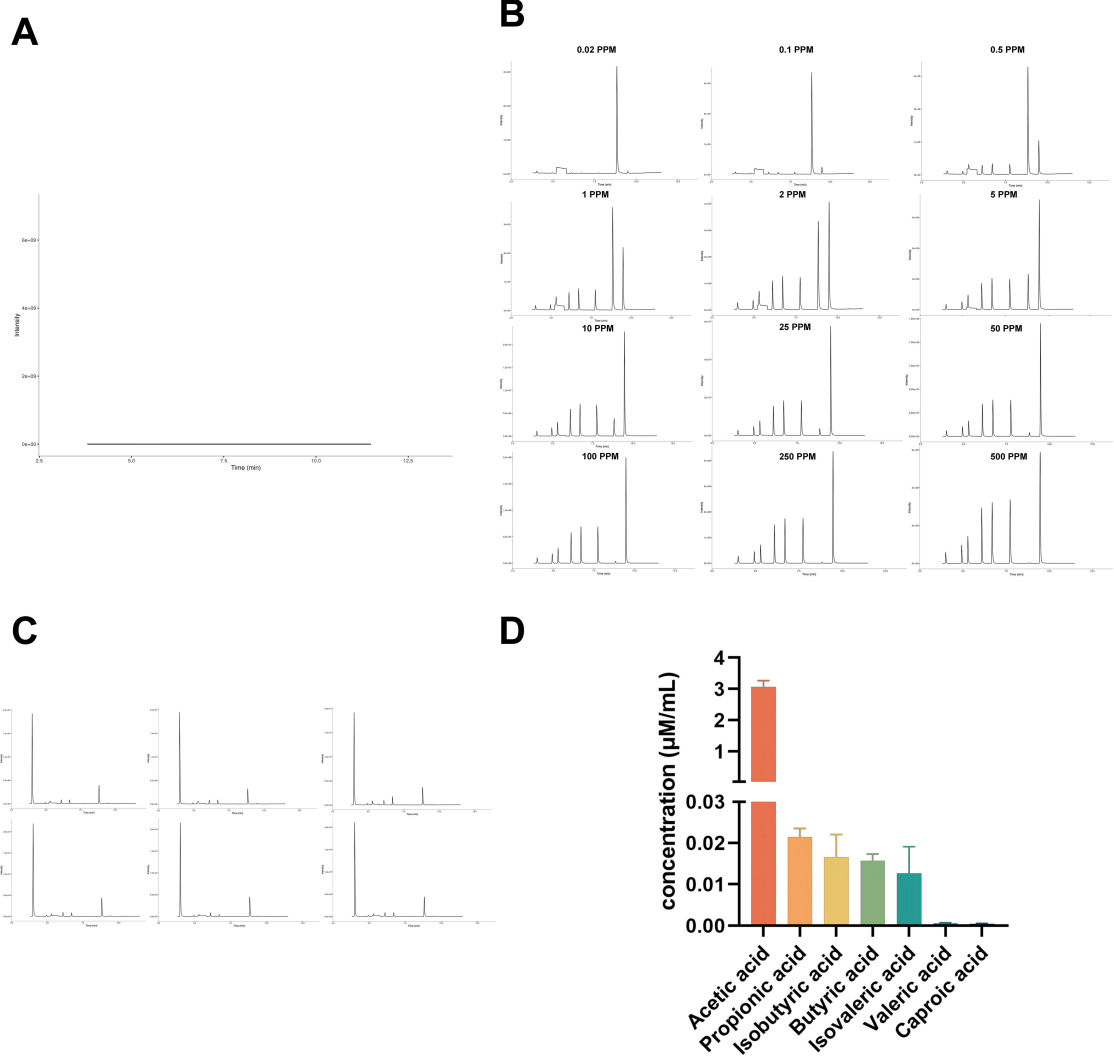
Results of the SCFA assay. (**A**) TIC of the reagent blank control sample. (**B**) TICs of mixed standard samples with concentrations of 0.02 ppm–500 ppm. (**C**) TIC of the *B. cereus* CL2 fermentation broth supernatant. (**D**) SCFA concentration in the CL2 supernatant (*n* = 6).

The total ion chromatogram (TIC) demonstrated the absence of spurious peaks in the reagent blank control sample (Fig. 9A), indicating a stable and error-free system with no false-positives. In the mixed sample of standards, the peaks of the internal standard and other standard substances were well-defined and distinguishable (Fig. 9B). This method exhibited stability with no false-negatives, which allows for the establishment of a standard regression curve.

The TIC of the supernatant from the *B. cereus* CL2 fermentation broth displayed a lack of interference from heterogeneous peaks, with good reproducibility of the samples (Fig. 9C). Quantification of SCFAs based on the TIC results from the CL2 supernatant revealed that CL2 could produce seven SCFAs (Fig. 9D). The concentrations of acetic, propionic, isobutyric, butyric, isovaleric, valeric, and caproic acids were 3.0638 ± 0.2045 µM/mL, 0.0215 ± 0.002 µM/mL, 0.0165 ± 0.0056 µM/mL, 0.0157 ± 0.0016 µM/mL, 0.0126 ± 0.0065 µM/mL, 0.00057 ± 0.00014 µM/mL, and 0.00044 ± 0.00011 µM/mL, respectively.

### Analysis of the colonic flora by 16S rDNA sequencing

**Fig 10:**
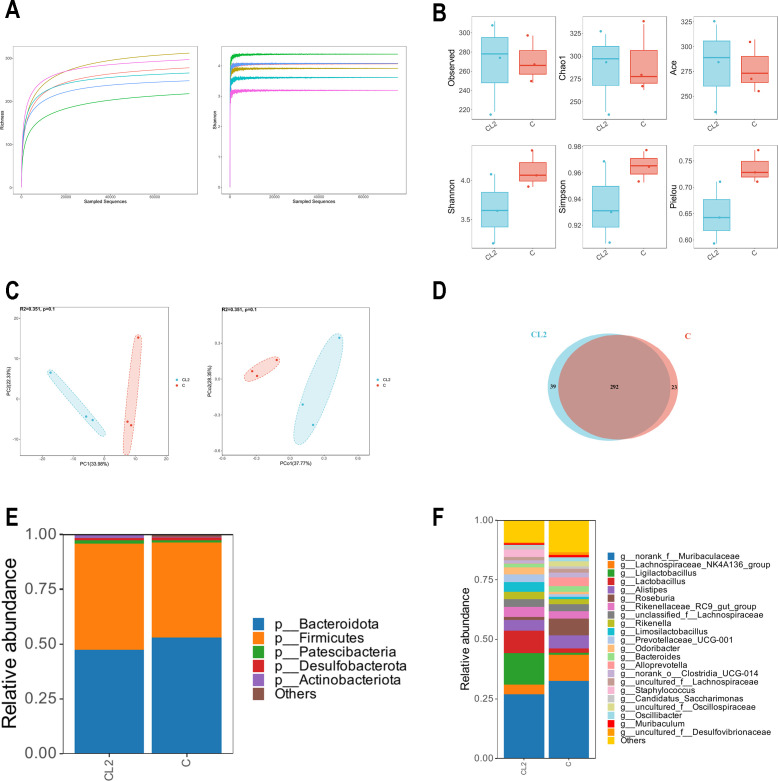
Effect of *B. cereus* CL2 on the intestinal flora in mice. The control group is denoted by C, and the test group is denoted by CL2. (**A**) Rarefaction curve and Shannon curve. (**B**) Alpha diversity analysis based on Chao1, ACE, Shannon, and Simpson indices. (**C**) Beta diversity analysis based on multivariate statistical methods, principal component analysis and principal coordinate analysis. (**D**) Venn diagram showing the overlapping OTUs between the two groups. (**E**) Mean relative abundances at the phylum level. (**F**) Mean relative abundances at the genus level.

The sequencing results revealed that as the number of reads increased, the rarefaction curve and Shannon‒Wiener curve gradually reached plateaus, indicating sufficient sequencing depth to reflect most of the microbial information in the samples (Fig. 10A). Alpha diversity analysis between the *B. cereus* gavage group and the control group demonstrated no significant differences in the Chao1, Ace, Shannon, and Simpson indices (*P* > 0.05) (Fig. 10B). PCA and PCoA exhibited good intragroup clustering with no significant distinction between the two groups (*P* > 0.05) (Fig. 10C). The Venn diagram illustrates that 292 OTUs were shared between the two groups, with 39 OTUs exclusive to the CL2 group and 23 OTUs exclusive to the control group (Fig. 10D).

These findings suggest that gavage of *B. cereus* CL2 did not significantly impact the abundance or diversity of the colonic flora in mice. Analysis at the phylum level (Fig. 10E) revealed a similar mean abundance of the intestinal flora between the two groups. However, there was a decrease in the abundance of *Bacteroidota* and an increase in the abundance of *Firmicutes* in the CL2 group compared to the control group. At the genus level, the abundance of *Lachnospiraceae* was reduced in the CL2 group, while *Ligilactobacillus* and *Lactobacillus* exhibited increased abundances compared to the control group (Fig. 10F).

## DISCUSSION

Cellulolytic bacteria are key to cellulose breakdown; they effectively enhance dietary fiber utilization and produce a variety of SCFAs to maintain intestinal health (13, 16, 17). In this study, we isolated three cellulolytic *B. cereus* strains from Kele pig feces. The cellulase activities of CL2 and CL4 are similar to those of the *Bacillus* sp. MKAL6 and the *Hymenobacter* sp. MKAL2 isolated from soil. Among the most suitable carbon sources, the cellulase activities of MKAL6 and MKAL2 were 190.30 U/mL and 78.87 U/mL, respectively (18). The CL3 is similar to the *Bacillus velezensis* M2 isolated from the gut of Min pigs. The cellulase activity of M2 in the optimum case was 41.18 U/mL (19). These findings indicated that CL2 and CL4 had stronger cellulolytic abilities and were identical to the efficient cellulolytic bacteria found in nature. Whole-genome sequencing revealed that more than 37% of the genes in the isolates are involved in metabolic processes, including energy, amino acid, carbohydrate, lipid and inorganic ion metabolism. These results are similar to those with *Bacillus* sp. DU-106, which was isolated from fermented yogurt (20). More than 35% of its genes were involved in metabolism, and this bacterium could efficiently produce lactate. In contrast, the functional genes of the isolates in this study were enriched in the cellulose metabolism pathway. This finding suggests a genetic basis for efficient cellulose breakdown and organic acid production in the isolates.

Some strains of *Bacillus* can produce toxins and transfer antibiotic resistance genes (21). In this study, three strains of *B. cereus* carried nonhemolytic enterotoxin genes and cytotoxin K genes. Nonhemolytic enterotoxin and cytotoxin K are common virulence factors in *Bacillus cereus*. They are often considered significant contributors to diarrhea, but their pathogenic mechanism in animal intestines remains unclear (22). Although they have been demonstrated to be cytotoxic *in vitro*, *B. cereus* carrying solely nonhemolytic enterotoxin genes is incapable of causing diarrhea or histopathological changes in pigs (23, 24). Therefore, cytotoxin K and nonhemolytic enterotoxins may not be the primary factors responsible for diarrhea, and further investigation is needed to assess their synergistic effects with other virulence factors (25). Additionally, isolates CL3 and CL4 carried the complete set of hemolysin BL genes and have demonstrated toxicity in animal tests. CL3 and CL4 caused weight loss in mice along with clinical symptoms such as bloody stool and diarrhea. The CL3 strain exhibited stronger virulence, leading to animal death. Conversely, the CL2 strain did not cause weight loss or histopathological changes in the mice. *Bacillus* sp. DU-106 also carried the hemolysin BL genes, but its toxin was expressed as an inactive protein due to an important amino acid change caused by a sec-type signal peptide (17, 26). Therefore, we considered hemolysin BL to be an important factor influencing the toxicity of *Bacillus cereus*. Plasmids are major factors in the pathogenicity and host interactions in *Bacillus*, and the major pathogenic plasmids in the *Bacillus cereus* group include pXO1 from *B. anthracis*, which carries structural genes for toxin proteins, and pCER270 from *B. cereus*, which encodes enzymatic components needed for the biosynthesis of the toxin cereulide (27). These plasmids and associated toxin genes were not detected in the isolates in this study. The drug susceptibility test results revealed that the isolates were susceptible to a wide range of commonly used antibiotics and resistant to penicillin and ampicillin. The observed results aligned with those of the predicted resistance gene analysis.

*B. cereus* commonly harbors multiple virulence genes and exhibits a complex pathogenic mechanism, which has led previous studies to predominantly focus on its contamination of food and the diseases it causes (28, 29). Recent studies have revealed that certain strains of *B. cereus* carrying virulence genes are nonpathogenic and exhibit beneficial effects in animal experiments. For instance, *B. toyonensis* BCT-7112T, a member of the *B. cereus* group, possesses nonhemolytic enterotoxin and hemolysin BL coding genes in its genome. However, supplementation with this strain was noncytotoxic, and this strain did not induce any pathogenic effects in animals. Notably, this strain has been employed as a feed additive for many years.^30^, ^31^ *B. cereus* HMPM18123 carried the complete hemolysin BL, nonhemolytic enterotoxin genes, cytotoxin K, and enterotoxin gene *entFM*. However, it exhibited immunomodulatory effects, preserved the integrity of intestinal barrier function, and mitigated inflammation in a mouse model of colitis induced by dextran sodium sulfate (32, 33). The application of *B. cereus* remains a topic of controversy, but its significant potential for industrial use and as a probiotic should not be overlooked. Further study is necessary to assess the safety and probiotic properties of different strains. In this study, the *B. cereus* CL2 grew rapidly and tolerated the simulated intestinal environment. The ability of a probiotic to reach the host intestinal tract and stably survive in it is crucial for eliciting its beneficial effects. Therefore, the growth performance and tolerance of probiotics are of paramount importance (34).

SCFA production is an important probiotic potency of bacteria. The study has indicated that *Bacillus clausii* T and *Lactobacillus reuteri* were acetic acid-producing probiotics, with acetic acid concentrations of 0.01 µM/mL and 0.011 µM/mL in their supernatants, and did not produce propionic acid (35). In contrast, the *B. cereus* CL2 in this study has a stronger acetic acid production capacity and produces a variety of SCFAs. The synergistic impact of multiple SCFAs contributes to a more comprehensive regulation of intestinal health (36). In another study, *Paenibacillus azoreducens* P8 had an efficient acetic acid production capacity. The average of the daily acetification rate was 13,554.92 µM/mL (37). The concentration of the CL2 strain is much lower than that of the P8 strain. Therefore, the application of *B. cereus* CL2 in industrial fermentation is not satisfactory and needs to be further investigated. Sequencing of the mouse intestinal flora revealed that gavage of *B. cereus* CL2 had no significant impact on the diversity and abundance of the intestinal microbiota. However, the CL2 group exhibited an increased abundance of *Firmicutes* and a decreased abundance of *Bacteroidota* at the phylum level. At the genus level, there was an increase in the abundance of *Ligilactobacillus* and *Lactobacillus. Firmicutes* play a crucial role in the breakdown of dietary fiber and serve as a communication pathway in the dietary fiber*–Firmicutes*–host axis within the animal intestine, and its metabolites are essential for maintaining overall health.^38^ *Ligilactobacillus* and *Lactobacillus* are extensively used probiotic genera in food and animal husbandry production. Numerous studies have demonstrated the probiotic properties of *Ligilactobacillus* and *Lactobacillus*, and an increased abundance of these genera can contribute to the promotion of intestinal health (39, 40).

In conclusion, the cellulolytic *B. cereus* CL2 exhibited a nonpathogenic effect in mice and adaptation of the intestinal environment. Furthermore, its cellulolytic properties make it highly efficient in the production of short-chain fatty acids. We consider *B. cereus* CL2 is a promising candidate for use as a commercial probiotic or in feed supplement.

## MATERIALS AND METHODS

### Sample collection

Fresh feces from healthy Kele pigs were collected from the Gaopo farm located in Guiyang City, Guizhou Province, China, into 50-mL aseptic screw pipes and delivered to the laboratory within 3 h.

### Isolation of the cellulolytic bacteria

Five milliliters of each GAM, TSB, PYG, R2A, RCM, Columbia, and BHI medium was prepared to which 250 µL of sterile defibrinated sheep blood was added, after which 0.1 g of feces from the center of the fecal pellet was added to each medium for 48 h of incubation at 37°C and 170 r/min. After gradient dilution, 100 µL of the dilution solution of each gradient (10^−2^–10^−6^) was evenly spread on CMC-Na agar medium (CMC-Na 10 g/L, tryptone 5 g/L, yeast extract 0.5 g/L, KH₂PO₄ 5 g/L, KCl 1.5 g/L, MgSO4 0.2 g/L, NaCl 5 g/L, and agar powder 20 g/L) and placed at 37°C for aerobic and anerobic incubation. The colonies were differentiated according to their morphology, and single colonies were picked for purification. This process was repeated several times until only colonies with the same morphology were present on the plate surface. Then, the strains were frozen using glycerol. The purified strain was inoculated in the GAM liquid medium (Coolaber Co., Ltd, China) and incubated at 37°C for 12 h for further detection. Cellulase activity was detected by using a cellulase activity kit (Beijing Solarbio Science & Technology Co., Ltd, China). One unit of cellulase activity (U) was defined as the amount of enzyme that catalyzed the breakdown of cellulose in the reaction system to obtain 1 µg of glucose per minute. The strains with high cellulase activity were screened for use.

### 16S rRNA sequencing

Bacterial DNA was extracted using the Bacterial Universal DNA Extraction Kit (Beijing Solarbio Science & Technology Co., Ltd, China), and PCR amplification was performed using 16S rRNA universal primers (27F: 5′-AGAG-TTTGATCCTGGCTCAG-3′, 1492R: 5′- GGTTA-CCTTGTTACGACTT-3′). The amplified products were sent to Sangon Biotech (Shanghai) Co., Ltd. for sequencing. The sequencing results were compared with those in BLAST in the NCBI to identify the homology of the strains, and the sequences of the strains with higher homology were selected to construct a phylogenetic tree using the neighbor-joining method with MEGA 11 software.

### Whole-genome sequencing and annotation

Whole-genome sequencing was performed using the Illumina HiSeq sequencing technology, the raw data obtained were quality-trimmedd by Trimmomatic after quality assessment by FastQC, and the sequencing data were spliced using SPAdes. The contig obtained from splicing was supplemented with GAP using GapFiller, and sequence correction was performed using PrInSeS-G. Gene elements were predicted using Prokka, and repetitive sequences in the genome were identified using RepeatMasker. Gene protein sequences were aligned with VFDB, CARD, and databases using NCBI Blast to annotate virulence and resistance genes. The gene protein sequences were aligned with the COG database and KAAS annotation to obtain GO functional information as well as KEGG pathway information. The gene sequences were aligned with the CAZy database using HMMER3 to analyze the carbohydrate-active enzymes (20, 41).

### Animal safety assessment

Forty 6–8-week-old female SPF BALB/C mice purchased from Slack Jingda Laboratory Animal Co., Ltd. (Hunan, China) were randomly divided into four groups (*n* = 10), all of which were fed a standard mouse diet and housed at 24°C ± 1°C with 50%–70% humidity on a 12-h light cycle. After 1 week of acclimatization, the control group was gavaged with 100 µL of sterile PBS daily, and the experimental groups were gavaged with 100 µL of *B. cereus* CL2, CL3, or CL4 at a concentration of 1 × 10^9^ CFU/mL for 28 d. The health status of the mice was observed daily, and the body weights of the mice were measured every 7 d. Mice that had been starved for 12 h were anesthetized with carbon dioxide inhalation, and blood was collected for routine blood testing. Then, the mice were sacrificed; the colon contents were collected for analysis of the colonic flora; and colon, liver, and spleen tissues were collected for pathological observations.

### Biological characteristics

#### General characteristics

Isolates were inoculated into LB agar medium (Beijing Solarbio Science & Technology Co., Ltd, China) to observe the colony characteristics, and the bacterial morphology was observed by Gram staining. The absorbance value of the bacterial solution at 600 nm was adjusted to 1. Two milliliters of the solution was added to 100 mL of LB liquid medium (Beijing Solarbio Science & Technology Co., Ltd, China) for incubation, and the OD_600_ value was measured by taking samples every 4 h over a total of 28 h. Growth curves were plotted using the results. Five milliliters of sterile PBS containing 0.3% bile salt was prepared. Then, 0.05 g of pepsin (Beijing Solarbio Science & Technology Co., Ltd, China) was added to 5 mL of sterile PBS, and the pH was adjusted to 3 with 1 mol/mL hydrochloric acid solution to prepare the artificial gastric fluid. Trypsin (0.05 g, Beijing Solarbio Science & Technology Co., Ltd, China) and 0.034 g of Na_2_HPO_4_ were added to 5 mL of sterile PBS to prepare the artificial intestinal fluid. Sterile PBS was used as a blank control. Then, 100 µL of the bacteria solution with OD_600_ = 1 was added to each solution, and the number of viable bacteria was detected after being placed at 37°C for 3 h.

First, 100 µL of the bacterial solution with an optical density OD_600_ = 1 was inoculated onto the surface of the LB agar medium. Then, a drug-sensitive paper (Hang Zhou Microbial Reagent Co., Ltd, China) was placed on the agar surface. The plates were incubated at 37°C for 12 h to observe bacterial inhibition, and the diameter of the inhibition zone was measured. The results were analyzed following the Performance Standards for Antimicrobial Susceptibility Testing (CLSI)M100-ED32.

#### Detection of short-chain fatty acids

*B. cereus* CL2 was inoculated in the GAM medium for 12 h of fermentation culture followed by centrifugation at 10,000 × *g* for 10 min, after which the supernatant was taken as the sample. A 100-mg/mL mixed standard stock solution of six SCFAs (acetic acid, propionic acid, isobutyric acid, butyric acid, isovaleric acid, and valeric acid) and a 100-mg/mL caproic acid stock solution were prepared with water and ether, respectively. A series of six SCFAs and caproic acid working solutions were prepared by appropriately diluting the standard stock solutions. A 75-µg/mL internal standard (IS) solution containing 4-methylvaleric acid was similarly prepared with ether. A twelve-point calibration curve was made by adding 220 µL of the working solutions, which contained 200 µL of each of the six acid working solutions from the series and 20 µL of each of the caproic acid working solutions from the series, 100 µL of 15% phosphoric acid, 20 µL of the 75 µg/ml IS solution, and 260 µL of ether covering a range from 0.02 to 500 µg/mL (0.02, 0.1, 0.5, 1, 2, 5, 10, 25, 50, 100, 250, and 500 µg/mL).

Samples were diluted twofold, and the diluted samples were extracted with 50 µL of 15% phosphoric acid and 10 µL of 75 µg/mL 4-methylvaleric acid solution as the IS and 140 µL of ether. Subsequently, the samples were centrifuged at 10,000 × *g* and 4°C for 10 min after vortexing for 1 min, and the supernatant was transferred to a vial prior to GC‒MS analysis. GC analysis was performed on a Trace 1300 gas chromatograph (Thermo Fisher Scientific, USA). Mass spectrometric detection of the metabolites was performed on an ISQ 7000 instrument (Thermo Fisher Scientific, USA) (42, 43).

### Analysis of the colonic flora by 16S rDNA sequencing

Microbial DNA was extracted from the colon content samples using an E.Z.N.A. soil DNA Kit (Omega Biotek, USA), and the V3–V4 hypervariable regions of the bacterial 16S rRNA gene were amplified with the primers 338F (5′-ACTCCTACGGGAGGCAGCAG-3′) and 806R (5′-GGACTACHVGGGTWTCTAAT-3′) by a thermocycler PCR system, respectively (GeneAmp 9700, USA). The PCR products were recovered using a 2% agarose gel and purified using the AxyPrep DNA Gel Extraction Kit (Axygen Biosciences, USA), and the DNA libraries were constructed by Illumina sequencing. Sequencing data were quality-controlled using fastp software, and OTU clustering of sequences was performed using Vsearch software (version 2.22.1) based on 97% similarity to obtain the OTUs and feature lists. The OTU sequences were annotated for species taxonomy using the RDP classifier (version 2.13). Beta diversity was calculated using the Bray‒Curtis distance to obtain beta diversity, and PCoA (principal coordinates analysis) and PERMANOVA (replacement multivariate analysis of variance) were performed based on the aforementioned distance matrix. Nonparametric rank-sum tests were used to detect differences in the microbial communities between groups, and correlations between specific species were analyzed by Spearman’s rank correlation (33, 44).

### Statistical analysis

All the data in this study were derived from at least three independent replicates, and all the data are reported as mean ± SD (standard deviation). Statistical analysis software (SPSS 25.0) was employed to conduct the correlation analysis of the results, and the differences among groups were compared by one-way analysis of variance, where *P* < 0.05 indicated that the differences were statistically significant. The statistical histogram graphs in this experiment were drawn by GraphPad Prism 8.0.

## Data Availability

The whole-genome sequencing and metagenomic data associated with the article have been uploaded to the NCBI SRA database under accession numbers PRJNA1076172 and PRJNA1076159, respectively.

## SUPPLEMENTAL MATERIAL

Table S1 spectrum.03267-23-s0001.xlsx COG blast results.

Table S2 spectrum.03267-23-s0002.xlsx KEGG annotations.

Table S3 spectrum.03267-23-s0003.xlsx Annotations of carbohydrate-active enzymes.

ASM does not own the copyrights to Supplemental Material that may be linked to, or accessed through, an article. The authors have granted ASM a non-exclusive, world-wide license to publish the Supplemental Material files. Please contact the corresponding author directly for reuse.

## References

[1] Hill C, Guarner F, Reid G, Gibson GR, Merenstein DJ, Pot B, Morelli L, Canani RB, Flint HJ, Salminen S, Calder PC, Sanders ME. 2014. Expert consensus document. The International scientific association for probiotics and prebiotics consensus statement on the scope and appropriate use of the term probiotic. Nat Rev Gastroenterol Hepatol 11:506–514. doi:10.1038/nrgastro.2014.66 24912386

[2] Doron S, Snydman DR. 2015. Risk and safety of probiotics. Clin Infect Dis 60:S129–S34. doi:10.1093/cid/civ085 25922398 PMC4490230

[3] Sanders ME, Merenstein DJ, Reid G, Gibson GR, Rastall RA. 2019. Probiotics and prebiotics in intestinal health and disease: from biology to the clinic. Nat Rev Gastroenterol Hepatol 16:605–616. doi:10.1038/s41575-019-0173-3 31296969

[4] O’Toole PW, Marchesi JR, Hill C. 2017. Next-generation probiotics: the spectrum from probiotics to live biotherapeutics. Nat Microbiol 2:17057. doi:10.1038/nmicrobiol.2017.57 28440276

[5] Ullah M, Rizwan M, Raza A, Zhao X, Sun Y, Gul S, Waheed MI, Khan MN, Gul A, Jan SU, Huang C. 2023. Comparative genomic and functional characterization of Lactobacillus casei group (LCG) probiotic strains isolated from traditional yogurts by next-generation sequencing. PJZ 55:1565–1573. doi:10.17582/journal.pjz/20210711190719

[6] Han Y, Ling Q, Wu L, Wang X, Wang Z, Chen J, Zheng Z, Zhou Z, Jia L, Li L, Wang B. 2023. Akkermansia muciniphila inhibits nonalcoholic steatohepatitis by orchestrating TLR2-activated? dt17 cell and macrophage polarization. GUT MICROBES 15:2221485. doi:10.1080/19490976.2023.2221485 37345844 PMC10288935

[7] Song WS, Jo SH, Lee JS, Kwon JE, Park JH, Kim YR, Baek JH, Kim MG, Kwon SY, Kim YG. 2023. Multiomics analysis reveals the biological effects of live Roseburia intestinalis as a high-butyrate-producing bacterium in human intestinal epithelial cells. Biotechnol J 18:e2300180. doi:10.1002/biot.202300180 37596881

[8] Froidurot A, Julliand V. 2022. Cellulolytic bacteria in the large intestine of mammals. Gut Microbes 14:2031694. doi:10.1080/19490976.2022.2031694 35184689 PMC8865330

[9] Cani PD. 2016. Interactions between gut microbes and host cells control gut barrier and metabolism. Int J Obes Suppl 6:S28–S31. doi:10.1038/ijosup.2016.6 28685027 PMC5485881

[10] Liu W, Luo X, Tang J, Mo Q, Zhong H, Zhang H, Feng F. 2021. A bridge for short-chain fatty acids to affect inflammatory bowel disease, type 1 diabetes, and non-alcoholic fatty liver disease positively: by changing gut barrier. Eur J Nutr 60:2317–2330. doi:10.1007/s00394-020-02431-w 33180143

[11] Fukuda S, Toh H, Hase K, Oshima K, Nakanishi Y, Yoshimura K, Tobe T, Clarke JM, Topping DL, Suzuki T, Taylor TD, Itoh K, Kikuchi J, Morita H, Hattori M, Ohno H. 2011. Bifidobacteria can protect from enteropathogenic infection through production of acetate. Nature 469:543–547. doi:10.1038/nature09646 21270894

[12] van der Hee B, Wells JM. 2021. Microbial regulation of host physiology by short-chain fatty acids. Trends Microbiol 29:700–712. doi:10.1016/j.tim.2021.02.001 33674141

[13] Parada Venegas D, De la Fuente MK, Landskron G, González MJ, Quera R, Dijkstra G, Harmsen HJM, Faber KN, Hermoso MA. 2019. Short chain fatty acids (SCFAs)-mediated gut epithelial and immune regulation and its relevance for inflammatory bowel diseases. Front Immunol 10:277. doi:10.3389/fimmu.2019.00277 30915065 PMC6421268

[14] Park J, Kim M, Kang SG, Jannasch AH, Cooper B, Patterson J, Kim CH. 2015. Short-chain fatty acids induce both effector and regulatory T cells by suppression of histone deacetylases and regulation of the mTOR-S6K pathway. Mucosal Immunol 8:80–93. doi:10.1038/mi.2014.44 24917457 PMC4263689

[15] Brooks L, Viardot A, Tsakmaki A, Stolarczyk E, Howard JK, Cani PD, Everard A, Sleeth ML, Psichas A, Anastasovskaj J, Bell JD, Bell-Anderson K, Mackay CR, Ghatei MA, Bloom SR, Frost G, Bewick GA. 2016. Fermentable carbohydrate stimulates FFAR2-dependent colonic PYY cell expansion to increase satiety. Mol Metab 6:48–60. doi:10.1016/j.molmet.2016.10.011 28123937 PMC5220466

[16] Chambers ES, Morrison DJ, Frost G. 2015. Control of appetite and energy intake by SCFA: what are the potential underlying mechanisms? Proc Nutr Soc 74:328–336. doi:10.1017/S0029665114001657 25497601

[17] Liao SF, Nyachoti M. 2017. Using probiotics to improve swine gut health and nutrient utilization. Anim Nutr 3:331–343. doi:10.1016/j.aninu.2017.06.007 29767089 PMC5941265

[18] Mokale Kognou AL, Chio C, Khatiwada JR, Shrestha S, Chen X, Han S, Li H, Jiang Z-H, Xu CC, Qin W. 2022. Characterization of cellulose-degrading bacteria isolated from soil and the optimization of their culture conditions for cellulase production. Appl Biochem Biotechnol 194:5060–5082. doi:10.1007/s12010-022-04002-7 35687308

[19] Li F, Xie Y, Gao X, Shan M, Sun C, Niu YD, Shan A. 2020. Screening of cellulose degradation bacteria from min pigs and optimization of its cellulase production. Electronic Journal of Biotechnology 48:29–35. doi:10.1016/j.ejbt.2020.09.001

[20] Li P, Tian WN, Jiang Z, Liang ZH, Wu XY, Du B. 2018. Genomic characterization and probiotic potency of Bacillus sp. DU-106, a highly effective producer of L-lactic acid isolated from fermented yogurt. Front Microbiol 9:2216. doi:10.3389/fmicb.2018.02216 30294310 PMC6158304

[21] Lee NK, Kim WS, Paik HD. 2019. Bacillus strains as human probiotics: characterization, safety, microbiome, and probiotic carrier. Food Sci Biotechnol 28:1297–1305. doi:10.1007/s10068-019-00691-9 31695928 PMC6811671

[22] Dietrich R, Jessberger N, Ehling-Schulz M, Märtlbauer E, Granum PE. 2021. The food poisoning toxins of Bacillus cereus. Toxins 13:98. doi:10.3390/toxins13020098 33525722 PMC7911051

[23] Koné KM, Hinnekens P, Jovanovic J, Rajkovic A, Mahillon J. 2021. New insights into the potential cytotoxic role of Bacillus cytotoxicus cytotoxin K-1. Toxins 13:698. doi:10.3390/toxins13100698 34678991 PMC8540763

[24] Trapecar M, Leouffre T, Faure M, Jensen HE, Granum PE, Cencic A, Hardy SP. 2011. The use of a porcine intestinal cell model system for evaluating the food safety risk of Bacillus cereus probiotics and the implications for assessing enterotoxigenicity. APMIS 119:877–884. doi:10.1111/j.1600-0463.2011.02797.x 22085364

[25] Castiaux V, Liu X, Delbrassinne L, Mahillon J. 2015. Is cytotoxin K from Bacillus cereus a bona fide enterotoxin? Int J Food Microbiol 211:79–85. doi:10.1016/j.ijfoodmicro.2015.06.020 26186121

[26] Ouhib-Jacobs O, Lindley ND, Schmitt P, Clavel T. 2009. Fructose and glucose mediates enterotoxin production and anaerobic metabolism of Bacillus cereus ATCC14579^T^. J Appl Microbiol 107:821–829. doi:10.1111/j.1365-2672.2009.04254.x 19302315

[27] Ehling-Schulz M, Lereclus D, Koehler TM. 2019. The Bacillus cereus group: Bacillus species with pathogenic potential. Microbiol Spectr 7. doi:10.1128/microbiolspec.GPP3-0032-2018 PMC6530592 31111815

[28] Zhu K, Hölzel CS, Cui YF, Mayer R, Wang Y, Dietrich R, Didier A, Bassitta R, Märtlbauer E, Ding SY. 2016. Probiotic Bacillus cereus strains, a potential risk for public health in China. Front Microbiol 7:718. doi:10.3389/fmicb.2016.00718 27242738 PMC4876114

[29] Rasko DA, Altherr MR, Han CS, Ravel J. 2005. Genomics of the Bacillus cereus group of organisms. FEMS Microbiol Rev 29:303–329. doi:10.1016/j.femsre.2004.12.005 15808746

[30] Jiménez G, Blanch AR, Tamames J, Rosselló-Mora R. 2013. Complete genome sequence of Bacillus toyonensis BCT-7112t, the active ingredient of the feed additive preparation toyocerin. Genome Announc 1:e01080-13. doi:10.1128/genomeA.01080-13 24356843 PMC3868867

[31] Williams LD, Burdock GA, Jiménez G, Castillo M. 2009. Literature review on the safety of toyocerin, a non-toxigenic and non-pathogenic Bacillus cereus var. toyoi preparation. Regul Toxicol Pharmacol 55:236–246. doi:10.1016/j.yrtph.2009.07.009 19631708

[32] Xu YF, Sheng KL, Wang YZ. 2023. Identiflcation of a heat-resistant strain of Bacillus cereus and evaluation of its efflcacy in alleviating inflammatory bowel disease. Food Science 44:173–180. doi:10.7506/spkx1002-6630-20220316-176

[33] Sheng KL, Xu YF, Kong XW, Wang JM, Zha XD, Wang YZ. 2021. Probiotic Bacillus cereus alleviates dextran sulfate sodium-induced colitis in mice through improvement of the intestinal barrier function, anti-inflammation, and gut microbiota modulation. J Agric Food Chem 69:14810–14823. doi:10.1021/acs.jafc.1c03375 34677958

[34] Ayyash MM, Abdalla AK, AlKalbani NS, Baig MA, Turner MS, Liu S-Q, Shah NP. 2021. Invited review: characterization of new probiotics from dairy and nondairy products—insights into acid tolerance, bile metabolism and tolerance, and adhesion capability. J Dairy Sci 104:8363–8379. doi:10.3168/jds.2021-20398 33934857

[35] Calvigioni M, Bertolini A, Codini S, Mazzantini D, Panattoni A, Massimino M, Celandroni F, Zucchi R, Saba A, Ghelardi E. 2023. HPLC-MS-MS quantification of short-chain fatty acids actively secreted by probiotic strains. Front Microbiol 14:1124144. doi:10.3389/fmicb.2023.1124144 36937254 PMC10020375

[36] Fusco W, Lorenzo MB, Cintoni M, Porcari S, Rinninella E, Kaitsas F, Lener E, Mele MC, Gasbarrini A, Collado MC, Cammarota G, Ianiro G. 2023. Short-chain fatty-acid-producing bacteria: key components of the human gut microbiota. Nutrients 15:2211. doi:10.3390/nu15092211 37432351 PMC10180739

[37] Krusong W, La China S, Pothimon R, Gullo M. 2022. Defining Paenibacillus azoreducens (P8) and Acetobacter pasteurianus (UMCC 2951) strains performances in producing acetic acid. Front Microbiol 13:991688. doi:10.3389/fmicb.2022.991688 36466629 PMC9712953

[38] Sun YG, Zhang SS, Nie QX, He HJ, Tan HZ, Geng F, Ji HH, Hu JL, Nie SP. 2022. Gut firmicutes: relationship with dietary fiber and role in host homeostasis. Crit Rev Food Sci Nutr 63:1–16. doi:10.1080/10408398.2022.2098249 35822206

[39] Balasubramanian B, Soundharrajan I, Al-Dhabi NA, Vijayaraghavan P, Balasubramanian K, Valan Arasu M, Choi KC. 2021. Probiotic characteristics of ligilactobacillus salivarius AS22 isolated from sheep dung and its application in corn-fox tail millet silage. Applied Sciences 11:9447. doi:10.3390/app11209447

[40] Liu DD, Gu CT. 2019. Lactobacillus pingfangensis sp. nov., Lactobacillus daoliensis sp. nov., Lactobacillus nangangensis sp. nov., Lactobacillus daowaiensis sp. nov., Lactobacillus dongliensis sp. nov., Lactobacillus songbeiensis sp. nov. and Lactobacillus kaifaensis sp. nov., isolated from traditional chinese pickle. Int J Syst Evol Microbiol:3237–3247. doi:10.1099/ijsem.0.003619 31361212

[41] Hu X, Qian YJ, Gao ZP, Li GY, Fu FH, Guo JJ, Shan Y. 2023. Safety evaluation and whole genome sequencing for revealing the ability of Penicillium oxalicum WX-209 to safely and effectively degrade citrus segments. Food Science and Human Wellness 12:2369–2380. doi:10.1016/j.fshw.2023.03.005

[42] Zhang SM, Wang HB, Zhu MJ. 2019. A sensitive GC/MS detection method for analyzing microbial metabolites short chain fatty acids in fecal and serum samples. Talanta 196:249–254. doi:10.1016/j.talanta.2018.12.049 30683360

[43] Tsukahara T, Matsukawa N, Tomonaga S, Inoue R, Ushida K, Ochiai K. 2014. High-sensitivity detection of short-chain fatty acids in porcine Ileal, cecal, portal and abdominal blood by gas chromatography-mass spectrometry. Anim Sci J 85:494–498. doi:10.1111/asj.12188 24612389

[44] Bai J, Zhao J, Al-Ansi W, Wang J, Xue L, Liu J, Wang Y, Fan M, Qian H, Li Y, Wang L. 2021. Oat β-glucan alleviates DSS-induced colitis via regulating gut microbiota metabolism in mice. Food Funct 12:8976–8993. doi:10.1039/d1fo01446c 34382058

